# Depression Socialization in Early Adolescent Friendships: The Role of Baseline Depressive Symptoms and Autonomous Functioning

**DOI:** 10.1007/s10964-023-01776-9

**Published:** 2023-05-03

**Authors:** Esther L. Bernasco, Jolien van der Graaff, Stefanie A. Nelemans, Tessa M. L. Kaufman, Susan Branje

**Affiliations:** Department of Youth and Family, Utrecht University, Utrecht, Netherlands

**Keywords:** Depressive symptoms, Individual differences, Autonomy, Peer influence, Friendship, Adolescence

## Abstract

There is mixed evidence for depression socialization, a process by which friends affect each other’s level of depressive symptoms. The current study examined whether adolescents’ baseline depressive symptoms and three dimensions of autonomous functioning (autonomy, peer resistance, and friend adaptation) make adolescents more or less sensitive to depression socialization, and how these dimensions of autonomous functioning were connected. In this preregistered, two-wave longitudinal study, participants completed questionnaires on depressive symptoms, autonomy, and peer resistance and participated in a task to assess friend adaptation. Participants were 416 Dutch adolescents (*M*_*age*_ = 11.60, 52.8% girls) across 230 close friend dyads. In contrast to expectations, results showed no significant depression socialization nor significant moderation. Furthermore, autonomy and peer resistance were related but distinct constructs, and not related to friend adaptation. These findings suggest that there is no depression socialization in early adolescence, regardless of level of autonomous functioning.

## Introduction

During adolescence, friends become increasingly important. Adolescents spend more time with peers and turn to peers for support more often (e.g., Helsen et al., 2000), and close friendships become more intimate (Selfhout et al., 2009). Whereas close friendships have many positive effects, such as providing social support (Cornwell, 2003) and protecting against maladjustment (Waldrip et al., 2008), several studies have also shown *depression socialization* among adolescents, which is a process by which friends’ (non-clinical) depressive symptoms predict increases in adolescents’ depressive symptoms (e.g., Giletta et al., 2011). Because there is inconsistency in findings on depression socialization (Neal & Veenstra, 2021), the current study tested whether some adolescents may be more sensitive to the effects of friends’ depressive symptoms on their own depressive symptoms than others, depending on baseline depressive symptoms and three dimensions of autonomous functioning.

### Depression Socialization

Friends tend to be similar in their level of depressive symptoms (Stone et al., 2013), partly because adolescents tend to select friends who are similar to them and break off friendships with peers who are more dissimilar (i.e., selection processes; Van Zalk et al., 2010a) and partly because friends affect each other and become more similar over time (i.e., socialization processes; Van Zalk et al., 2010a). The interpersonal theory of depression emphasizes socialization processes and states that relationships with others can cause or exacerbate symptoms of depression (Coyne, 1976). Adolescents with high levels of depressive symptoms may, for example, induce negative moods in their friends when they show many negative and few positive emotions or when they use maladaptive interaction and coping styles, such as repeated reassurance seeking, negative feedback seeking and co-rumination. In addition, friends may imitate these maladaptive interaction behaviors and coping styles, such as co-rumination (Schwartz-Mette & Rose, 2012) and depressogenic attributional style (Stevens & Prinstein, 2005). As such, depression socialization between close friends may occur through mechanisms of mood contagion or imitation. These effects may be most pronounced during early adolescence, when youth are particularly vulnerable to the effects of peer influence (Laursen & Veenstra, 2021).

Dyadic studies on socialization between close friends indeed showed that friend depressive symptoms predict adolescent depressive symptoms, while controlling for previous depressive symptoms when averaging all of one adolescent’s close friends (Goodwin, Mrug, Borch, & Cillessen, 2012), and in both reciprocated and unreciprocated friend dyads (Stevens & Prinstein, 2005). Some studies report depression socialization to be equal in size across age and gender, when comparing early and mid-adolescence (Schwartz-Mette & Smith, 2018) or when comparing children and adolescence (Schwartz-Mette & Rose, 2012), whereas others only found depression socialization in girls (Giletta et al., 2011) or found that gender interacted with other personal characteristics, such as popularity or social anxiety, to predict sensitivity to depression socialization (Prinstein, 2007). However, most studies on depression socialization do not examine other potential moderators beside age or gender. Further evidence for depression socialization comes from network studies examining socialization within the broader peer group, although the evidence is also mixed: A recent systematic review on depression socialization in adolescence using stochastic actor-oriented models showed that 4 out of 7 studies found significant socialization effects, but was not able to explain why some studies do find depression socialization and others do not, and whether there are moderators that may explain the mixed findings (Neal & Veenstra, 2021). The current study improved on the previous literature by taking into account that not all adolescents may be equally sensitive to depression socialization. More specifically, this study aimed to examine the moderating effect of baseline depressive symptoms and autonomous functioning.

### Individual Differences in Depression Socialization

According to the integrated environmental sensitivity framework (Pluess, 2015), some people are more sensitive to their environment than others. An individual’s sensitivity to depression socialization may depend, for example, on adolescents’ initial level of depressive symptoms. Adolescents with lower levels of depressive symptoms may be more psychologically resilient against adversity and other negative influences (Poole et al., 2017), and may be less likely to be affected by friends who score higher on depressive symptoms. Instead, they may have the resources to support friends who experience difficulties. Adolescents with higher levels of depressive symptoms may be more vulnerable and sensitive to their friends’ depressive symptoms as they likely are less able to cope with others’ emotional difficulties compared to adolescents with lower levels of depressive symptoms (Orzechowska et al., 2013). In line with these ideas, depression socialization within friendship dyads has been found to be stronger for adolescents who reported higher levels of personal distress (Prinstein, 2007). Also, in a study of adolescents and their romantic partners, having a partner with high levels of sadness was particularly detrimental for adolescents who also reported more sadness. It is likely that similar effects occur within close friendships, where adolescents who are more vulnerable in terms of higher levels of depressive symptoms may be more sensitive to depression socialization. However, this has not been examined in friendship dyads. The current study tested whether adolescents’ baseline depressive symptoms may make them more vulnerable to friends’ depressive symptoms.

Furthermore, adolescents’ vulnerability to depression socialization may depend on their level of autonomous functioning (Allen et al., 2006), which can be defined as one’s perception of freedom to behave and believe based on intrinsic motivation rather than external pressures (Soenens et al., 2018). Previous research has shown that higher levels observed autonomy in mother-child interactions are associated with lower vulnerability to peer substance use socialization (Allen et al., 2012), whereas higher levels of observed autonomy-relatedness behaviors in friend interactions increased vulnerability to peer substance use (Allen et al., 2022). As autonomous functioning may be expressed differently across different contexts, the current study distinguishes three dimensions of autonomous functioning that differ in terms of context specificity, ranging from the broad context of overall perceived autonomy to the narrow context of specific situations in a close friendship (Steinberg & Silverberg, 1986): General autonomy, peer resistance, and friend adaptation.

*General autonomy* is a dimension of autonomous functioning in the broadest sense and refers to one’s general sense of volitional functioning (Soenens et al., 2018) or the extent to which people follow their own wants, needs, and beliefs rather than complying with external wishes and other people’s beliefs. Adolescents who are more autonomous may be less affected by friends’ negative mood and interaction styles and thereby less sensitive to depression socialization. Adolescents who have a stronger self-concept clarity, or sense of who they are, which is one aspect of autonomy, were found less likely to be affected by friends’ socialization of delinquency (Levey et al., 2019). Similarly, adolescents who perceive to have a stronger choice in how they act, feel and think may be less sensitive to depression socialization. To the authors’ knowledge, the current study is the first to test whether autonomy may affect vulnerability to depression socialization.

Whereas general autonomy applies to different social contexts, *peer resistance* can be seen as a dimension of autonomous functioning that takes place specifically within the peer group context and can be defined as the extent to which adolescents can resist pressure from their peers. More specifically, it refers to being able to stick to one’s own opinion and refuse when an adolescent is asked or pressured by friends to something they do not want to do or would not do if they were alone (Santor et al., 2000), including but not limited to risk-taking and rule-breaking behavior. Adolescents who are better able to resist peers and stay true to their own wishes, ideas, and needs, may be better able to distinguish between the self and the other in friendships and thereby be less sensitive to the negative mood and maladaptive coping and interaction styles of friends that are associated with depression socialization (Schwartz-Mette & Rose, 2012). As such, it is possible that adolescents who are more resistant to peer pressure are less sensitive to depression socialization. However, just like general autonomy, this has not been tested before.

Whereas general autonomy and peer resistance as dimensions of autonomous functioning operate in a broader social context*, friend adaptation* focuses on the context of dyadic relationship in which the depression socialization is assumed to take place. It can be defined as the extent to which adolescents accept their friends’ viewpoints in interactions, rather than maintaining their own viewpoints. This type of autonomous functioning mirrors the way adolescents develop autonomy from parents (Allen et al., 2006). Friend adaptation, like social conformity, can be overt or internal (Sowden et al., 2018): Overt compliance relates to the extent to which adolescents overtly agree with their friend (e.g., to keep the peace, to make a decision, to be liked), whereas internal acceptance relates to the extent to which adolescents internally accept their friends’ opinion as their own (e.g., they are convinced that they were wrong before or change their mind). Adolescents who showed more overt compliance in a conversation task were more sensitive to friends’ socialization of externalizing problem behavior (Allen et al., 2006). Possibly, higher levels of overt compliance make adolescents more sensitive to not just socialization of observable behaviors, such as externalizing problems, but also to depression socialization, although this has not been tested before.

The literature on internal acceptance is scarce, and to the authors’ knowledge there are no studies on internal acceptance in the friendship context, in relation to depressive symptoms or to other socialization processes. Studies on peer influence typically examine how behavior changes when adolescents are in the presence of their friends (i.e., overt compliance), but it remains unclear whether these changes persist when they are alone (i.e., internal acceptance). Yet, to fully understand the effects of peer influence beyond the interaction, it is important to understand to what extent adolescents internalize peer influence. On the one hand, internal acceptance may reflect a lack of autonomous functioning, perhaps even more so than overt compliance, as it can be seen as instability in one’s beliefs. Based on this view, it can be expected that adolescents who score high on internal acceptance are more sensitive to their friends’ influence, including the effect of their depressive symptoms. On the other hand, internal acceptance may reflect higher levels of autonomous functioning, when it is viewed as the autonomous decision to change one’s mind. As such, it would be expected that internal acceptance does not make adolescents more sensitive to depression socialization, or even less sensitive. Therefore, the current study explores the effects of both overt compliance and internal acceptance separately.

Although general autonomy, peer resistance, and friend adaptation stem from different theoretical backgrounds, they can all be seen as different dimensions of autonomous functioning, which operate in different contexts and may increase or decrease adolescents’ sensitivity to their friends’ depressive symptoms (e.g., Allen et al., 2006). Although there is some overlap in the sense that they all measure some type of sensitivity to others, they tap into difference processes: Having a general sense of autonomy does not necessarily translate to never complying to peer pressure, and pressure from the general group can have a different effect on individuals than differences in opinion with friends. Also, while complying with or conforming to friend influence may indicate a lack of autonomy, it could also reflect a voluntary, autonomous decision to comply with friends in that moment, and might have benefits as well, such as higher relationship quality (Allen et al., 2022). Thus, it can be expected that there may be differences between (some of) the constructs, although they have not yet been studied together. Therefore, the current study aimed to examine to what extent these dimensions are different and overlapping.

Although the main interest of this study is the potential moderating effect of autonomous functioning in depression socialization, it is possible that general autonomy, peer resistance, and friend adaptation also directly affect depressive symptoms. Autonomous functioning is necessary for healthy development, and a lack of autonomous functioning may result in psychosocial problems. Previous studies have shown that low general autonomy is associated with low self-esteem, negative affect (Patrick et al., 2007), and depressive symptoms (Van der Giessen et al., 2014), low levels of peer resistance were associated with an increase in depressive symptoms, and overt friend adaptation is related to an increase in depressive symptoms (Allen et al., 2006). The current study tested whether adolescents who score higher on autonomous functioning report lower levels of depressive symptoms.

## Current Study

**Fig. 1:**
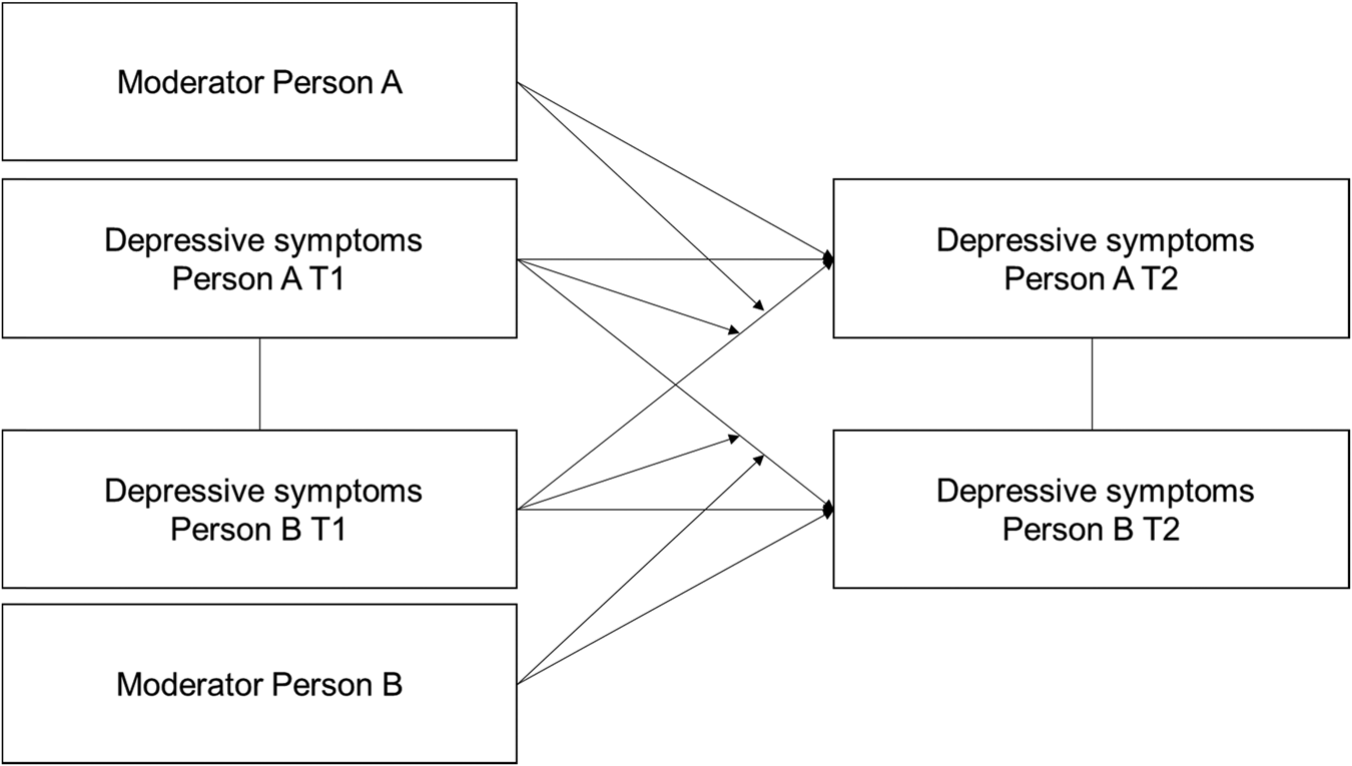
A visual representation of the hypothesized effects

Previous research has provided mixed evidence for depression socialization, and studies have often failed to take into account that some adolescents may be more sensitive to depression socialization than others. Therefore, the current study aimed to test whether depression socialization depends on adolescents’ baseline depressive symptoms and autonomous functioning by examining three dimensions of autonomous functioning that may be distinct but related: General autonomy, peer resistance, and friend adaptation. First, this study tested the associations between these three dimensions of autonomous functioning. It was expected that these dimensions are moderately correlated, reflecting that they are distinct but related constructs. Next, the study aimed to replicate previous work on dyadic depression socialization. In line with previous research, it was expected that friend depressive symptoms are positively associated with adolescent depressive symptoms six months later, controlling for earlier adolescent depressive symptoms. Last, this study aimed to test whether depression socialization depended on baseline depressive symptoms and autonomous functioning. It was expected that depression socialization is stronger for adolescents who report more depressive symptoms at baseline. It was also expected that general autonomy and peer resistance are negatively associated with adolescent depressive symptoms, and overt compliance and internal acceptance are positively associated with depressive symptoms six months later. Also, it was expected that friend depression socialization is stronger for adolescents who (a) report less autonomy, (b) report less peer resistance, or (c) show more overt and internal friend adaptation. The hypothesized model was pre-registered (https://osf.io/8quxv/) and is illustrated in Fig. 1.

## Methods

### Procedure

This study included the first two waves of the longitudinal multi-informant INTRANSITION study on identity and autonomy development across school transitions. The majority of the participants (83.3%) started in the fall of 2019, and a second smaller cohort (16.7%) started a year later. In the first wave, participants and their friends participated in a conversation task together during a home visit and completed questionnaires on depressive symptoms, general autonomy, and peer resistance. In the second wave, which took place six months later, they again reported on their depressive symptoms. Participants and their friends both received €10 for each completed questionnaire set at each wave. They received an additional €10 for participating in observation tasks and could receive a bonus €5 if they participated in all four planned waves. All participants and one of their parents signed informed consent before participation. This procedure was approved by the Faculty Ethics Review Board of the Faculty of Social & Behavioural Sciences of Utrecht University.

### Participants

Adolescents were recruited by contacting schools in the Netherlands. In schools that decided to cooperate, information about the study was sent to adolescents and their parents via the school and the researchers pitched the project for adolescents and their parents. Information was distributed among adolescents who were in the final year of primary school at the start of the study, and adolescents were asked to contact the researchers if they were interested in participating. There was no evidence for nesting within schools: In 85% of the cases, there were five or less participants per school, and when there were more these participants were mostly from different classrooms.

Participants were 416 adolescents across 230 close-friend dyads (195 dyads started in 2019 and 35 started in 2020) ranging from age 10 to 14 (*M* = 11.60, *SD* = 0.49). Of all adolescents, 47.0% were boys, 52.8% were girls, and 0.3% were non-binary/other. Socioeconomic status (SES) was measured by asking participants to rate their general situation compared to the national situation on a scale from 1 (much worse) to 10 (much better). Self-reported SES was relatively high, with a mean of 7.8 (*SD* = 1.14) and 89.4% rating their SES 7 or higher. The majority of the sample reported a Dutch ethnicity (95.8%) and was born in the Netherlands (96.4%).

Ten participants were chosen as close friend twice, and 33 adolescents participated without a close friend in both waves. Furthermore, for 55 dyads (23.1%) data was missing because one or both of the adolescents did not participate in one of the waves. Of adolescents who participated with a friend, 77 (39.1%) were all-boy dyads, 93 (47.2%) were all-girl dyads, 11 (5.6%) were mixed-gender dyads, and 16 (8.1%) were dyads with some missing gender information. In Wave 2, participants reported that their friendships had lasted on average 5.6 years (*SD* = 40.01), and 94.2% indicated that their friendship had lasted more than 6 months. Participants were free to choose anyone as their close friend; friend selection was not restricted to classmates or reciprocal (best) friends.

### Measures

#### Depressive symptoms

Depressive symptoms were assessed in both waves using the mean of the Major Depressive Disorder subscale of the self-report Revised Child Anxiety and Depression Scale (Chorpita et al., 2000). Adolescents and their friends indicated for each of 10 statements how often they applied to them on a scale ranging from 1 (never) to 4 (always). Example items are “I feel sad or empty” and “I don’t have the energy to do things.” Reliability of the depressive symptoms subscale was good, Cronbach’s α = 0.82 (T1)—0.87 (T2).

#### General autonomy

General autonomy was assessed in Wave 1 using the mean of the Perceived Choice scale from the Perceived Choice and Awareness Scale (Sheldon, 1995). Adolescents and their friends rated on 5 items to what extent they generally felt free to make their own choices in life, on a scale from 1 (not true at all) to 7 (completely true). Example items are: “I always feel like I choose the things I do myself” and “I do what I do because it interests me”. Reliability of the scale was good, Cronbach’s α = 0.84.

#### Peer resistance

Peer resistance was assessed in Wave 1 using the mean of 14 items that were based on the Resistance to Peer Influence questionnaire (Steinberg & Monahan, 2007). Questions pertained to pressure from the broader friend group, not limited to best friends or school friends. Adolescents and their friends indicated to what extent the 14 items applied to them on a scale from 1 (completely disagree) to 5 (completely agree). The scale included items such as “I would break the rules if my friends say they do too” and “I often go along with my friends, because I’m scared they will be unhappy if I don’t”, which were reverse coded to reflect peer resistance. Reliability of the scale was excellent, Cronbach’s α = 0.91.

#### Friend adaptation

Friend adaptation was assessed in Wave 1 using a dilemma task that was an extension of the Mars Task and the Sinking Ship Dilemma (Allen et al., 2006). In the current dilemma task, participants were asked to imagine that their ship was about to sink and they could bring 7 items (out of 12 alternatives) to a deserted island. Like in the original task, first adolescents and friends made the decision individually (baseline). Next, adolescents and their friends had to reach agreement on the 7 items together (together-phase) to assess to what extent participants went along with their friend (overt compliance). The current study added a third phase, in which participants made the decision alone again (post-phase) to assess to what extent adolescents changed their own opinion based on the dyadic discussion (internal acceptance). To create more variance, participants were asked in each phase to rank the chosen items in a top-7, instead of just choosing seven items which was done in the original version of the task. In this ranking, 1 indicated the highest priority, and 7 indicated the lowest priority. Non-chosen items were recoded as ‘8’. Participants could not see their friends’ or their own responses from the earlier phases at any point. The items were relatively similar in usefulness (e.g., pocketknife, food cans, sunscreen) to promote discussion about the decisions.

An *overt compliance* score was calculated for each adolescent and friend as overt opinion change (participants’ difference between the baseline and the together-phase) divided by the initial disagreement (difference between the two friends at baseline). An *internal acceptance* score was calculated for each adolescent and friend as internal opinion change (participants’ difference between the baseline and the post-phase) divided by the initial disagreement between the two friends. In these calculations, “difference” reflects the sum of absolute difference of the 12 item positions. Friend adaptation scores that were obtained using this novel calculation method are significantly correlated (overt compliance: *r* = 0.47, internal acceptance: *r* = 0.44, *p*s < 0.001) with scores based on the original calculation method, but have the advantage that (1) items do not need to be removed when both friends chose it, (2) dyads do not need to be removed when friends choose the same 7 items, and (3) it takes into account shifts in priority for certain items to be chosen (rather than only taking into account whether an item was chosen or not). In previous studies, overt compliance measured in this task was predictive of negative peer influence one year later and made adolescents more sensitive to peer drug and alcohol use (Allen et al., 2006), suggesting its validity to measure sensitivity to friend influence.

### Analysis

Descriptive statistics and correlations were performed in IBM SPSS Statistics 25 and socialization models were modeled in M*plus* version 8.7 (Muthén & Muthén, 1998–2017), using the MplusAutomation package (Hallquist & Wiley, 2018) in RStudio (R Core Team, 2021). Alpha = 0.05 (two-tailed) was used for significance testing, and standardized estimates were interpreted (Cohen, 1977). Little’s MCAR test showed that data were missing at random, χ^2^ (41) = 49.03, *p* = 0.182, and missing data, including friend data for 33 adolescents participating without a friend, were handled using full information maximum likelihood estimation (Muthén & Muthén, 1998–2017). All continuous variables were standardized across the whole sample (targets and friends), and interaction terms were calculated based on these standardized variables. Models were only interpreted if they met two of the following three fit requirements: CFI ≥ 0.90, RMSEA ≤ 0.08, SRMR ≤ 0.08 (Byrne, 2012). Dimensions of autonomous functioning were interpreted as related but distinct constructs if they were moderately correlated to each other (i.e., 0.20 < *r* < 0.50; Cohen, 1977).

To test depression socialization and moderation, six actor-partner interdependence models (APIMs; Kenny, 1996) were fit. Dyadic socialization research typically uses this model to asses socialization (e.g., Schwartz-Mette & Smith, 2018). An advantage of APIM, particularly in the context of studying socialization processes, is that bidirectional effects can be modeled simultaneously (i.e., socialization of person A to person B and socialization of person B to person A). In an APIM, each variable is measured for each person in the dyad. The main model, which was the basic depression socialization model, included four variables: Depressive symptoms of the target and friend on T1 and T2. The models include so-called ‘actor effects’ (e.g., depressive symptoms target T1 → depressive symptoms target T2), ‘partner effects’ (e.g., depressive symptoms target T1 → depressive symptoms friend T2) and concurrent associations (e.g., depressive symptoms target T1 ↔ depressive symptoms friend T1). Note that the effect of friend depressive symptoms on target depressive symptoms and the effect of target depressive symptoms on friend depressive symptoms were modeled simultaneously (see Fig. 1).

In each dyad, it was randomized who was assigned the role of “target” and the role of “friend”. After randomization, target adolescents and friends did not differ on gender, χ^2^ (2) = 1.18, *p* = 0.556, depressive symptoms (T1 and T2), or any of the four dimensions of autonomous functioning, |t | < 1.38, *p* > 0.170, suggesting that targets and friends could be seen as so-called “indistinguishable dyads”. This means that there is no specific feature that differentiates one friend from the other, as would be the case for gender in heterosexual romantic relationships, for example. Therefore, parameters of actor paths and partner paths, as well as means, intercepts, variances, and residuals were constrained to be equal for both friends (Olsen & Kenny, 2006). Wald tests suggested that these parameters could indeed be constrained to be equal in all models (*p*s ≥ 0.157), except for the covariance between the main effect of autonomy and the interaction: Freeing the parameter resulted in significantly better model fit, Wald(1) = 5.02, *p* = 0.025.^1^

Ten adolescents were in the dataset twice because they were chosen as friend by two participants, or because they were chosen by someone as friend but chose another friend for themselves. This dependency in the data was taken into account by applying a correction for the nested structure in the final analyses (using the TYPE = COMPLEX command).

Depression socialization was reflected in significant partner effects in the APIM. To test whether baseline depressive symptoms moderated depression socialization, an interaction term (target depressive symptoms T1 * friend depressive symptoms T1) was added to the basic socialization model. This interaction term predicted both adolescent and friend depressive symptoms T2. To assess the main and moderator effects of the different dimensions of autonomous functioning (i.e., autonomy, peer resistance, overt compliance, and internal acceptance), a separate APIM was fit for each moderator. Effects of the adolescent moderator on adolescent depressive symptoms, and interaction effects of the adolescent moderator and friend depressive symptoms on adolescent depressive symptoms were added to the basic socialization model, resulting in two additional estimated paths per model. Any significant interaction effects were further probed using simple slopes analysis to test at what levels of moderator the socialization effect was significant.

## Results

### Descriptive Statistics

Descriptive statistics collapsed across adolescents and friends are displayed in Table 1. Depressive symptoms at both time points were negatively correlated with autonomy and peer resistance at T1. There were significant intra-dyad correlations for peer resistance (at T1) and depressive symptoms (T2), suggesting some degree of similarity between friends in terms of peer resistance and depressive symptoms.

**Table 1:** Descriptive statistics and correlations

Variable	M (SD)	Dyadic correlations	Bivariate correlations				
			1	2	3	4	5
1 Depressive symptoms T1	1.45 (0.37)	0.09					
2 Depressive symptoms T2	1.47 (0.41)	0.25*	0.61^***^				
3 Autonomy	5.63 (0.91)	0.03	−0.23^***^	−0.23^***^			
4 Peer resistance	4.17 (0.61)	0.18*	−0.29^***^	−0.36^***^	0.30^***^		
5 Overt compliance	0.67 (0.31)	−0.08	−0.02	−0.03	0.01	0.00	
6 Internal acceptance	0.63 (0.33)	0.05	0.00	−0.02	−0.05	−0.03	0.81^***^

*Note*. Dyadic correlations refer to the correlations between two friends within a dyad

*p < 0.05, ***p < 0.001

In line with expectations, the correlation between self-reported general autonomy and peer resistance was moderate in size (*r* = 0.30, *p* < 0.001), suggesting these are distinct but related constructs. In contrast to expectations, the friend adaptation measures, overt compliance and internal acceptance, were not significantly correlated with the self-reported measures of autonomous functioning, suggesting these are distinct constructs. The two measures of friend adaptation were strongly correlated with each other (*r* = 0.81, *p* < 0.001).

### Depression Socialization

Next, the six APIMs were fit. Model fit was satisfactory for all models (see Table 2). In contrast to the hypothesis, the basic depression socialization APIM revealed a nonsignificant partner effect (*p* = 0.102), indicating that there was no depression socialization. The actor effect was significant (β = 0.60, *p* < 0.001), suggesting stability of depressive symptoms for each dyad member over time. Furthermore, the within-time associations between depressive symptoms of the adolescent and friend were not significant at either T1 or T2 (*p*s > 0.265), indicating that there was also no similarity in depressive symptoms at each wave.

**Table 2:** Model fit statistics

Model	CFI	RMSEA [95% CI]	SRMR	AIC	χ^2^
Depression socialization	1.00	0.00 [0.00, 0.05]	0.03	1177.21	3.00(6), *p* = 0.81
Baseline depressive symptoms	1.00	0.00 [0.00, 0.03]	0.03	1635.43	3.20(8), *p* = 0.92
Autonomy	0.92	0.06 [0.02, 0.09]	0.08	2933.22	35.49(21), *p* = 0.02
Resistance	1.00	0.00 [0.00, 0.05]	0.07	2865.01	18.90(22), *p* = 0.65
Overt compliance	1.00	0.00 [0.00, 0.05]	0.08	3055.79	21.77(22), *p* = 0.47
Internal acceptance	1.00	0.00 [0.00, 0.03]	0.07	3104.42	15.24(22), *p* = 0.85

### Moderation by Depressive Symptoms

The moderating effect of actor baseline depressive symptoms on depression socialization was examined and was not significant, in contrast to expectations (*p* = 0.949). This suggests that depression socialization did not depend on baseline depressive symptoms.

### Moderation by Autonomous Functioning

To test the hypothesis that adolescents with more autonomous functioning experience less depressive symptoms and less depression socialization, main and interaction effects of autonomous functioning were examined separately for all four dimensions of autonomous functioning: General autonomy, peer resistance, overt compliance, and internal acceptance. Regarding general autonomy, in contrast to expectations there was no significant main effect of actor general autonomy T1 on actor depressive symptoms T2 (*p* = 0.544). In addition, the interaction of actor general autonomy with partner depressive symptoms T1 on actor depressive symptoms T2 was not significant (*p* = 0.879), suggesting that depression socialization did not depend on adolescent general autonomy. The T1 within-time association between actor depressive symptoms and actor autonomy was significant (β = −0.21, *p* < 0.001), indicating that general autonomy was negatively associated with depressive symptoms at T1.

Regarding peer resistance, in line with expectations, peer resistance T1 had a small but significant negative actor effect on depressive symptoms T2 (β = −0.08, *p* < 0.001), suggesting that adolescents who were more resistant to peer influence experienced less depressive symptoms six months later. In contrast to expectations, there was no significant interaction between actor peer resistance and partner depressive symptoms on actor depressive symptoms T2 (*p* = 0.628), indicating that depression socialization did not depend on adolescent peer resistance. The T1 within-time association between actor depressive symptoms and actor peer resistance was significant T1 (β = −0.30, *p* < 0.001).

Regarding friend adaptation, in contrast to expectations there was no significant main effect of overt compliance T1 on depressive symptoms T2 (*p* = 0.830), and no significant interaction of actor overt compliance T1 with partner depressive symptoms T1 on actor depressive symptoms T2 (*p* = 0.886). There was also no significant main effect of internal acceptance T1 on depressive symptoms T2 (*p* = 0.567), and no significant interaction of actor internal acceptance T1 with partner depressive symptoms T1 on actor depressive symptoms T2 (*p* = 0.750). These results show that depression socialization did not depend on either overt compliance or internal acceptance (See Tables 3–8 for all model estimates).

**Table 3:** Estimates for the basic depression socialization model

Path	β	S.E.
*Stability*		
DepSym A T1 → DepSym A T2 ^1^	0.60***	0.05
DepSym B T1 → DepSym B T2 ^1^	0.60***	0.05
*Depression socialization*		
DepSym A T1 → DepSym B T2 ^2^	0.11	0.07
DepSym B T1 → DepSym A T2 ^2^	0.11	0.07
*Within-time associations*		
DepSym A T1 ↔ DepSym B T1	0.13	0.11
DepSym A T2 ↔ DepSym B T2	0.01	0.10

*Note*. DepSym A = Depressive symptoms adolescent, DepSym B = Depressive symptoms friend. Paths with equal superscripts were constrained to be equal across friends

***p < 0.001

**Table 4:** Estimates for the model including moderation by baseline depression

Path	β	S.E.
*Stability*		
DepSym A T1 → DepSym A T2 ^1^	0.60***	0.05
DepSym B T1 → DepSym B T2 ^1^	0.60***	0.05
*Depression socialization*		
DepSym A T1 → DepSym B T2 ^2^	0.11	0.06
DepSym B T1 → DepSym A T2 ^2^	0.11	0.06
*Moderation by baseline depressive symptoms*		
DepSym A * DepSym B T1 → DepSym A T2 ^3^	0.01	0.09
DepSym A * DepSym B T1 → DepSym B T2 ^3^	0.01	0.09
*Within-time associations*		
DepSym A T1 ↔ DepSym B T1	0.13	0.11
DepSym A T2 ↔ DepSym B T2	0.01	0.10
DepSym A T1 ↔ DepSym A T1 * DepSym B T1 ^4^	0.23	0.16
DepSym B T1 ↔ DepSym A T1 * DepSym B T1 ^4^	0.23	0.16

*Note*. DepSym A = Depressive symptoms adolescent, DepSym B = Depressive symptoms friend. Paths with equal superscripts were constrained to be equal across friends

***p < 0.001

**Table 5:** Estimates for the model including moderation by general autonomy

Path	β	S.E.
*Stability*		
DepSym A T1 → DepSym A T2 ^1^	0.59***	0.05
DepSym B T1 → DepSym B T2 ^1^	0.59***	0.05
*Depression socialization*		
DepSym A T1 → DepSym B T2 ^2^	0.11	0.07
DepSym B T1 → DepSym A T2 ^2^	0.11	0.07
*Main effects*		
Auton. A → DepSym A T2 ^3^	−0.03	0.05
Auton. B → DepSym B T2 ^3^	−0.03	0.05
*Moderation effects*		
DepSym B T1 * Auton. A → DepSym A T2 ^4^	0.01	0.06
DepSym A T1 * Auton. B → DepSym B T2 ^4^	0.01	0.06
*Within-time associations*		
DepSym A T1 ↔ DepSym B T1	0.13	0.11
DepSym A T2 ↔ DepSym B T2	0.01	0.10
Auton. B ↔ Auton. A	0.07	0.09
DepSym B T1 * Auton. A ↔ DepSym A T1 * Auton. B	0.03	0.15
DepSym A T1 ↔ Auton. A ^5^	−0.21***	0.05
DepSym B T1 ↔ Auton. B ^5^	−0.21***	0.05
DepSym A T1 ↔ Auton. B ^6^	−0.03	0.06
DepSym B T1 ↔ Auton. A ^6^	−0.03	0.06
DepSym A T1 ↔ DepSym A T1 * Auton. B ^7^	0.08	0.10
DepSym B T1 ↔ DepSym B T1 * Auton. A ^7^	0.08	0.10
DepSym A T1 ↔ DepSym B T1 * Auton. A ^8^	0.00	0.05
DepSym B T1 ↔ DepSym A T1 * Auton. B ^8^	0.00	0.05
Auton. A ↔ DepSym A T1 * Auton. B ^9^	−0.19***	0.05
Auton. B ↔ DepSym B T1 * Auton. A ^9^	−0.19***	0.05
Auton. A ↔ DepSym B T1 * Auton. A	0.13	0.12
Auton. B ↔ DepSym A T1 * Auton. B	−0.37**	0.15

*Note*. DepSym A = Depressive symptoms adolescent, DepSym B = Depressive symptoms friend. Auton = Autonomy. Paths with equal superscripts were constrained to be equal

**p < 0.01, ***p < 0.001

**Table 6:** Estimates for the model including moderation by peer resistance

Path	β	S.E.
*Stability*		
DepSym A T1 → DepSym A T2 ^1^	0.54***	0.05
DepSym B T1 → DepSym B T2 ^1^	0.54***	0.05
*Depression socialization*		
DepSym A T1 → DepSym B T2 ^2^	0.11	0.06
DepSym B T1 → DepSym A T2 ^2^	0.11	0.06
*Main effects*		
Resist A → DepSym A T2 ^3^	−0.20**	0.05
Resist B → DepSym B T2 ^3^	−0.20**	0.05
*Moderation effects*		
DepSym B T1 * Resist A → DepSym A T2 ^4^	0.03	0.06
DepSym A T1 * Resist B → DepSym B T2 ^4^	0.03	0.06
*Within-time associations*		
DepSym A T1 ↔ DepSym B T1	0.12	0.11
DepSym A T2 ↔ DepSym B T2	0.02	0.10
Resist B ↔ Resist A	0.20**	0.10
DepSym A T1 * Resist B ↔ DepSym B T1 * Resist A	0.19	0.19
DepSym A T1 ↔ Resist A ^5^	−0.30***	0.06
DepSym B T1 ↔ Resist B ^5^	−0.30***	0.06
DepSym A T1 ↔ Resist B ^6^	−0.09	0.07
DepSym B T1 ↔ Resist A ^6^	−0.09	0.07
DepSym A T1 ↔ DepSym A T1 * Resist B ^7^	−0.25**	0.11
DepSym B T1 ↔ DepSym B T1 * Resist A ^7^	−0.25**	0.11
DepSym A T1 ↔ DepSym B T1 * Resist A ^8^	−0.11	0.10
DepSym B T1 ↔ DepSym A T1 * Resist B ^8^	−0.11	0.10
Resist A ↔ DepSym A T1 * Resist B ^9^	0.01	0.09
Resist B ↔ DepSym B T1 * Resist A ^9^	0.01	0.09
Resist A ↔ DepSym B T1 * Resist A ^10^	−0.05	0.11
Resist B ↔ DepSym A T1 * Resist B ^10^	−0.05	0.11

*Note*. DepSym A = Depressive symptoms adolescent, DepSym B = Depressive symptoms friend, Resist = peer resistance. Paths with equal superscripts were constrained to be equal

**p < 0.01, ***p < 0.001

**Table 7:** Estimates for the model including moderation by overt compliance

Path	β	S.E.
*Stability*		
DepSym A T1 → DepSym A T2 ^1^	0.60***	0.05
DepSym B T1 → DepSym B T2 ^1^	0.60***	0.05
*Depression socialization*		
DepSym A T1 → DepSym B T2 ^2^	0.11	0.07
DepSym B T1 → DepSym A T2 ^2^	0.11	0.07
*Main effects*		
Comp A → DepSym A T2 ^3^	−0.01	0.05
Comp B → DepSym B T2 ^3^	−0.01	0.05
*Moderation effects*		
DepSym B T1 * Comp A → DepSym A T2 ^4^	−0.01	0.06
DepSym A T1 * Comp B → DepSym B T2 ^4^	−0.01	0.06
*Within-time associations*		
DepSym A T1 ↔ DepSym B T1	0.13	0.11
DepSym A T2 ↔ DepSym B T2	0.01	0.10
Comp B ↔ Comp A	−0.09	0.21
DepSym A T1 * Comp B ↔ DepSym B T1 * Comp A	0.03	0.10
DepSym A T1 ↔ Comp A ^5^	−0.03	0.05
DepSym B T1 ↔ Comp B ^5^	−0.03	0.05
DepSym A T1 ↔ Comp B ^6^	0.00	0.04
DepSym B T1 ↔ Comp A ^6^	0.00	0.04
DepSym A T1 ↔ DepSym A T1 * Comp B ^7^	−0.05	0.09
DepSym B T1 ↔ DepSym B T1 * Comp A ^7^	−0.05	0.09
DepSym A T1 ↔ DepSym B T1 * Comp A ^8^	−0.12	0.07
DepSym B T1 ↔ DepSym A T1 * Comp B ^8^	−0.12	0.07
Comp A ↔ DepSym A T1 * Comp B ^9^	−0.06	0.12
Comp B ↔ DepSym B T1 * Comp A ^9^	−0.06	0.12
Comp A ↔ DepSym B T1 * Comp A ^10^	−0.20	0.14
Comp B ↔ DepSym A T1 * Comp B ^10^	−0.20	0.14

*Note*. DepSym A = Depressive symptoms adolescent, DepSym B = Depressive symptoms friend, Comp = Overt compliance. Paths with equal superscripts were constrained to be equal

***p < 0.001

**Table 8:** Estimates for the model including moderation by internal acceptance

Path	β	S.E.
*Stability*		
DepSym A T1 → DepSym A T2 ^1^	0.60***	0.05
DepSym B T1 → DepSym B T2 ^1^	0.60***	0.05
*Depression socialization*		
DepSym A T1 → DepSym B T2 ^2^	0.11	0.07
DepSym B T1 → DepSym A T2 ^2^	0.11	0.07
*Main effects*		
Accept A → DepSym A T2 ^3^	−0.03	0.05
Accept B → DepSym B T2 ^3^	−0.03	0.05
*Moderation effects*		
DepSym B T1 * Accept A → DepSym A T2 ^4^	−0.02	0.07
DepSym A T1 * Accept B → DepSym B T2 ^4^	−0.02	0.07
*Within-time associations*		
DepSym A T1 ↔ DepSym B T1	0.13	0.11
DepSym A T2 ↔ DepSym B T2	0.01	0.11
Accept B ↔ Accept A	0.05	0.18
DepSym B T1 * Accept A ↔ DepSym A T1 * Accept B	0.05	0.12
DepSym A T1 ↔ Accept A ^5^	0.00	0.05
DepSym B T1 ↔ Accept B ^5^	0.00	0.05
DepSym A T1 ↔ Accept B ^8^	0.00	0.05
DepSym B T1 ↔ Accept A ^8^	0.00	0.05
DepSym A T1 ↔ DepSym A T1 * Accept B ^6^	−0.09	0.10
DepSym B T1 ↔ DepSym B T1 * Accept A ^6^	−0.09	0.10
DepSym A T1 ↔ DepSym B T1 * Accept A ^7^	−0.06	0.07
DepSym B T1 ↔ DepSym A T1 * Accept B ^7^	−0.06	0.07
Accept A ↔ DepSym A T1 * Accept B ^9^	−0.13	0.10
Accept B ↔ DepSym B T1 * Accept A ^9^	−0.13	0.10
Accept A ↔ DepSym B T1 * Accept A ^10^	−0.14	0.13
Accept B ↔ DepSym A T1 * Accept B ^10^	−0.14	0.13

*Note*. DepSym A = Depressive symptoms adolescent, DepSym B = Depressive symptoms friend, Accept = internal acceptance. Paths with equal superscripts were constrained to be equal

***p < 0.001

### Sensitivity Analysis: COVID-19 effects

As part of the sample started before the COVID-19 pandemic (fall 2019), and part of them started during the pandemic (fall 2020), analyses were run separately for the 2019 cohort only. Results for this subsample (84.8% of the full sample) were not different from results on the full sample. Additionally, time spent with friends increased equally for both cohorts from T1 to T2, suggesting a developmental trend only. These findings suggest that the results were minimally impacted by the pandemic.

### Post-Hoc Analysis: Gender Differences

As some previous findings showed that depression socialization depended on gender (Giletta et al., 2011), multigroup analyses on gender (boys vs. girls) were performed. As adolescents were allowed to choose non-same-gender friends in the current study, only all-boy (*n* = 77) and all-girl dyads (*n* = 91) were included to make interpretation easier. In the baseline model all parameters were constrained to be equal across gender. Subsequently, it was tested whether freeing parameters of interest one-by-one significantly improved model fit using Wald tests.

Measurement invariance tests showed evidence for at least metric invariance between boys and girls for all constructs. This suggests equality of scaling for all the measures (i.e., the items for each measure load onto the specified latent variable in a similar manner and with similar magnitude) for boys and girls and that regression coefficients can be compared between boys and girls in subsequent multi-group analyses.

Results for the multigroup analyses revealed that depression socialization effects were not significantly different for boys and girls. In the peer resistance model, the moderating effect of peer resistance on depressive symptoms was significant for girls (β = 0.16, *p* = 0.004) but not for boys (β = −0.02, *p* = 0.773). However, simple slopes analysis probed at low (*M* – 1 *SD*) and high (*M* + 1 *SD*) levels of peer resistance showed that the effect of friend depressive symptoms on adolescent depressive symptoms (i.e., depression socialization) was significant for neither low peer resistance (*t* = −0.083, *p* = 0.934) nor high peer resistance (*t* = 0.201, *p* = 0.841). The moderation effects of baseline depressive symptoms, general autonomy, or friend adaptation did not differ significantly. It is also worth noting that in the basic depression socialization model, the T1 covariance between adolescent and friend depressive symptoms significantly differed between boys (β = −0.24, *p* = 0.035) and girls (β = 0.27, *p* = 0.040). This suggests that girls with higher levels of depressive symptoms also have friends with higher levels of depressive symptoms, whereas boys with higher levels of depressive symptoms have friends with lower levels of depressive symptoms.

## Discussion

Adolescents may experience more depressive symptoms over time if they have a close friend who experiences higher levels of depressive symptoms (van Zalk et al., 2010b), but evidence for this process of depression socialization within close adolescent friendships is mixed (e.g., Neal & Veenstra, 2021). The current study further explored depression socialization in early adolescence by testing whether depression socialization is affected by baseline depressive symptoms and different dimensions of autonomous functioning, as well as how different dimensions of autonomous functioning may differ or overlap. The current study found no evidence for either depression socialization or moderation of depression socialization by depressive symptoms or autonomous functioning. Furthermore, general autonomy was related to yet distinct from peer resistance, but these dimensions were not related to friend adaptation, suggesting that they may not share a common underlying basis.

### Depression Socialization

In contrast to expectations, the current study did not find evidence for depression socialization within close friend dyads in early adolescence. This expectation was based on the idea that depressed adolescents may affect their friends’ depressive symptoms through mood contagion (Bastiampillai et al., 2013) or maladaptive interaction styles such as co-rumination (Schwartz-Mette & Rose, 2012). Possibly, mood contagion en interaction styles do not directly affect depressive symptoms: Mood contagion may be limited to the periods spent with friends and not affect mood or even depressive symptoms on the long term. Possibly, interaction styles may be shared within dyadic interactions, but may mostly be a consequence rather than a precedent of depressive symptoms in either friend. The current study was comparable to previous work that did show evidence for depression socialization in most aspects related to sample (size, age, gender and ethnicity distribution), design (two waves, six to twelve months apart, actor-partner models), and measurement (self-report questionnaires). One important difference to most studies is that the current study did not require friendship reciprocity, although many friends in the current study were reciprocal friends, and reciprocity status does not affect depression socialization (Stevens & Prinstein, 2005). There may also be individual differences in depression socialization: For some adolescents, their depressive symptoms may be more visible than for others, and depression socialization in their friendships may be stronger than for adolescents who mask their depressive symptoms (e.g., highly intelligent adolescents; Jackson & Peterson, 2003). The lack of socialization effects in the current study indicates that depression socialization in early adolescence may not always occur and may depend on individual factors or the social context.

### Individual Differences in Depression Socialization

It was expected that adolescents with high levels of depressive symptoms would experience stronger depression socialization, as they might be less well-equipped to deal with friends’ depressive symptoms. In contrast to expectations, baseline depressive symptoms did not moderate depression socialization, suggesting that there was no depression socialization between close friends regardless of baseline levels of depressive symptoms. Possibly, there is individual variation not just in depression socialization, but also in the effects of individual characteristics, such as baseline depressive symptoms, on depression socialization. Some adolescents with higher levels of depressive symptoms may be more vulnerable to negative aspects in their environment, whereas other adolescents with higher levels of depressive symptoms may be more rigid. For example, patients with chronic depression have been found to be less responsive to treatment than patients with non-chronic depression (Seemüller et al., 2022). Perhaps no overall effect of baseline depressive symptoms was found because for some adolescents poorer mental health is associated with higher sensitivity, while for others it is associated with lower sensitivity. Furthermore, for adolescents with high levels of depressive symptoms their own (negative) perception of the friendship may more relevant than the level of depressive symptoms actually reported by their friends. Adolescents with depressive symptoms may be less likely to recognize and accept social support (Ren et al., 2018).

Regarding autonomous functioning, it was expected that adolescents who experience more autonomous functioning, and as such are less influenced by others’ opinions and behaviors, would be less affected by depressive symptoms of their close friends. In contrast to this hypothesis, the current study found no evidence for the moderating effect of either general autonomy, peer resistance, or friend adaptation on depression socialization, suggesting that there was no depression socialization, regardless of the levels of these subdimensions of autonomous functioning.

Although almost no predictive effects of autonomous functioning were found, general autonomy and peer resistance were concurrently associated with depressive symptoms. According to self-determination theory, the development of autonomy is crucial for healthy psychosocial development (Deci & Ryan, 1995), and a lack of autonomy has been concurrently (Eagleton et al., 2016) and prospectively (Allen et al., 2006) associated with depressive symptoms. In contrast to general autonomy and peer resistance, friend adaptation (both overt compliance and internal acceptance) was not associated with depressive symptoms either concurrently or predictively. Friend adaptation may be more beneficial than a lack of general autonomy or peer resistance as it can help to maintain friendships, but in itself it may not be indicative of better or poorer psychosocial functioning. Instead, it may depend on the content of the friendship: Adaptation to friends may be beneficial in the context of a positive, supportive friendship, but less beneficial in more negative friendships with high levels of co-rumination, for example. Alternatively, the different findings may also reflect the difference in methods: Self-reported measures tap into perception of autonomous functioning, whereas observational measures assess behavior. According to the current study, in particular adolescents’ own perception of their autonomy may be important for their mental well-being, and this perception is not necessarily related to actual behavior. Furthermore, adolescents with more depressive symptoms may perhaps perceive themselves as less autonomous than they behave in real-life situations, in line with the finding that depressive symptoms are associated with a lower sense of control (Steptoe et al., 2007). This may explain why friend adaptation did not only fail to predict depressive symptoms, but was not concurrently related to depressive symptoms either.

Possibly, depression socialization does not depend as much on individual factors, such as the ones examined in the current study, but rather on characteristics of the social context in which depression socialization takes place. For example, friendship quality may affect depression socialization, as it may reflect how much time friends spend together and co-ruminate with each other. Friends who are not as close may not as easily affect each other (Schwartz-Mette & Smith, 2018). In addition, depression socialization may depend on the content of interactions between friends. Friends who co-ruminate together may be more likely to socialize depressive symptoms than friends who do not (Schwartz-Mette & Rose, 2012). Furthermore, friendship context may matter. The current study only looked at dyads, but depression socialization may occur differently, or not at all, in the peer group (Giletta et al., 2012).

### Difference and Overlap in Dimensions of Autonomous Functioning

In line with expectations, general autonomy and peer resistance were moderately correlated, suggesting that they are related but distinct constructs. General autonomy stems from self-determination theory (Deci & Ryan, 1995), whereas peer resistance stems from the peer influence literature (e.g., Santor et al., 2000). They have been mostly studied separately in the past, with general autonomy usually in the context of identity and the autonomy-relatedness balance, and peer resistance in the context of externalizing problem behavior and peer group influence. The current study provides preliminary evidence that they share a common basis and that there might be merit to studying them together. Yet, the moderate correlation also suggests that they are not the same and that autonomous functioning is a multidimensional construct. Autonomy may differ across context, which is supported by the finding that at the onset of adolescence, parental autonomy increases whereas peer autonomy first decreases and then increases again during adolescence (Steinberg & Monahan, 2007).

Furthermore, overt compliance and internal acceptance were highly correlated with each other, indicating that adolescents who were more likely to sway in a compromise situation were also more likely to be internally swayed. In contrast to expectations, the two types of friend adaptation, overt compliance and internal acceptance, were not related to the other dimensions of autonomous functioning: General autonomy and peer resistance. This suggests that friend adaptation (both overt compliance and internal acceptance) taps into different aspects of autonomous functioning than general autonomy and peer resistance do. This may be related to the idea of volition and independence as two orthogonal domains of autonomous functioning (Van Petegem et al., 2013). In the context of this study, this would mean that some adolescents comply with friends because they depend on their friends (i.e. dependence) and others comply because they actively decide to do so (i.e., volition). This also raises questions on the meaning of both overt compliance and internal acceptance as dimensions of autonomous functioning. Scoring high on overt and internal acceptance may reflect that adolescents have an uncertain identity and are easily swayed, but it may also mean that their friends had better arguments to convince them. Future studies should further explore the interplay between autonomous functioning and peer influence while distinguishing overt compliance and internal acceptance, both in the friend context and in other contexts.

### Gender Differences

Post-hoc analyses on the effect of gender showed that, although the socialization effect was not different for boys and girls, dyadic similarity significantly differed. Girls with more depressive symptoms tended to be friends with girls who also scored high on depressive symptoms, whereas boys who score high on depressive symptoms tend to be friends with boys with low levels on depressive symptoms. This may indicate that boys and girls use different processes to choose and maintain their friendships, and is in line with the finding that friendships of boys and girls tend to differ in terms of shared activities and self-disclosure (Rose, 2007). Most relevantly, girls tend to engage more in problem talk and co-rumination with their friends than boys (Rose, 2002). It should be noted that these conclusions are based on post-hoc analyses in smaller samples, so future research should not only replicate this finding, but also explore why boys and girls show these opposite patterns.

Beside this finding of (dis)similarity, no gender differences were found. As gender differences in constructs such as friendship behaviors (Rose & Rudolph, 2006) and depressive symptoms (Nolen-Hoeksema & Girgus, 1994) increase with age, it is possible that differences between boys and girls were not as pronounced in the current sample of youth in early adolescence. Another study in a slightly older sample found that depression socialization was only present for girls, for example (Giletta et al., 2011).

### Strengths and Limitations

A strength of this study was that it approached autonomous functioning as a multifaceted construct that may differ across contexts and may make an individual more or less sensitive to other forms of influence, such as depression socialization. Another strength of the study is the use of longitudinal dyadic data of adolescents and their close friends. This made it possible to longitudinally test socialization effects for both friend and target simultaneously. Lastly, an important strength of this study was the use of multi-method data, rather than relying solely on self-report. Adolescents with depressive symptoms are more likely to hold negative views about themselves and report more negatively about their autonomous functioning as well. Using observational data in addition to self-report questionnaire may provide a fuller picture.

However, this study has some limitations as well. First, it is not possible to definitively conclude whether the differences between friend adaptation and the other two dimensions of autonomous functioning were due to differences in measurement or because they are unrelated constructs. Furthermore, the level of depressive symptoms was relatively low as the study included a community sample. Results may differ in a sample with higher levels of depressive symptoms, such as an older sample or a clinical sample. These samples are likely to show more variability in depressive symptoms, whereas the current sample had relatively low variability on the higher end of the depressive symptoms spectrum.

Future research may further disentangle which groups of youth may be more vulnerable or resilient to depression socialization, for example using person-centered approaches. Furthermore, alternative processes of friend similarity may be at play. Another process that might affect friend similarity is depression mitigation, which involves decreases in depressive symptoms in the more depressed friend (Kiuru et al., 2012). This protective alternative process could be the topic of further investigations.

Post-hoc analyses and some previous research suggest that gender may be important to study. The current study did not have enough power to draw firm conclusions about gender, but future research should aim to increase sample size in order to test gender effects. Alternatively, research could focus on girls only, as girls tend to have more depressive symptoms, as well as more co-rumination (Rose, 2002), which may underlie depression socialization (Schwartz-Mette & Rose, 2012) and may affect depressive symptoms in girls only (Bastin et al., 2018).

## Conclusion

Previous research has shown some evidence for depression socialization within adolescent friendships, but results were mixed. The current study aimed to test whether differences in sensitivity to depression socialization can be explained by baseline depressive symptoms and autonomous functioning in early adolescence. Results showed no evidence for dyadic depression socialization, nor for individual differences in this socialization effect depending on level of depressive symptoms or three dimensions of autonomous functioning: General autonomy, peer resistance, and friend adaptation. Future research should continue to explore why some studies find depression socialization and others do not, or why some adolescents are sensitive to depression socialization and others are not. Furthermore, this study showed that general autonomy and peer resistance are related but distinct constructs. They have been mostly studied separately in the past, with general autonomy usually in the context of identity and self-determination theory, and peer resistance in the context of externalizing problem behavior and peer group influence. The current study provides preliminary evidence that they share a common basis.

## Footnotes

1 Two other Wald tests were significant: Freeing means and variances of internal acceptance worsened (rather than improved) model fit, Wald(4) 18.26, *p* = 0.001. Freeing main and moderation effects of autonomy significantly improved model fit (Wald(2) = 6.28, *p* = 0.043), but model fit of the constrained model was already acceptable. For reasons of parsimony these parameters were not freed.
